# Efficacy and safety of sepetaprost in patients with primary open-angle glaucoma or ocular hypertension: results from the randomised, phase 3 ANGEL-J1 study

**DOI:** 10.1007/s10384-026-01350-3

**Published:** 2026-06-16

**Authors:** Masaru Inatani, Akiyasu Kanamori, Maya Inai, Toshihiro Ikeda, Raymund Angeles

**Affiliations:** 1 Department of Ophthalmology, Faculty of Medical Sciences, University of Fukui, Yoshida, Japan; 2 Division of Ophthalmology, Department of Surgery, Kobe University Graduate School of Medicine, Kobe, Japan; 3 Kanamori Eye Clinic, Akashi, Japan; 4 Santen Pharmaceutical Co., Ltd., Osaka, Japan; 5 Santen Inc., 2100 Powell Street, Suite 800, Emeryville, CA 94608 USA

**Keywords:** Dual FP/EP3 receptor agonist, Intraocular pressure, Ocular hypertension, Primary open-angle glaucoma, Sepetaprost

## Abstract

**Purpose:**

To evaluate the efficacy and safety of sepetaprost, a novel dual FP/EP3 receptor agonist, in patients with primary open-angle glaucoma (POAG) or ocular hypertension (OHT).

**Study design:**

ANGEL-J1 (NCT05495061) was a phase 3, randomised, investigator-masked, active-controlled, parallel-group, non-inferiority study across 49 sites in Japan.

**Methods:**

Adults with POAG or OHT were randomised 1:1 to topical sepetaprost 0.002% or latanoprost 0.005%, once daily for 3 months. The primary endpoint was change in mean diurnal intraocular pressure (IOP) from baseline at week 4 (non-inferiority margin, 1.5 mmHg); a key secondary endpoint was mean change in IOP from baseline over 3 months. Safety outcomes included the incidence of adverse drug reactions (ADRs).

**Results:**

In total, 325 patients were randomised to sepetaprost (n=162) or latanoprost (n=163). In mixed model for repeated measures analyses, ANGEL-J1 met its primary endpoint; the least-squares mean (± standard error) change in mean diurnal IOP from baseline at week 4 was −5.77±0.16 mmHg in the sepetaprost group, which was non-inferior to −6.10±0.16 mmHg in the latanoprost group (least-squares mean difference, 0.32 mmHg [95% confidence interval, −0.12, 0.77]). Non-inferiority for the key secondary endpoint was also established, with comparable mean reductions in IOP between groups maintained over 3 months. ADRs occurred in 59 patients (36.4%) receiving sepetaprost and 33 (20.2%) receiving latanoprost; the most common ADR was conjunctival hyperaemia (29.6% and 8.6%, respectively; mostly mild in severity).

**Conclusion:**

In patients with POAG or OHT, sepetaprost demonstrated IOP-lowering efficacy that was non-inferior to latanoprost, and an acceptable safety profile.

**Supplementary Information:**

The online version contains supplementary material available at 10.1007/s10384-026-01350-3.

## Introduction

Glaucoma is the leading cause of irreversible blindness worldwide [1, 2]. Almost 7.5 million cases of glaucoma were estimated globally in 2019 [3]; in 2020, 3.6 million cases of blindness and 4.1 million cases of vision impairment were attributed to glaucoma [2, 4]. In Japan, the Tajimi study estimates the prevalence of glaucoma at 5.0% among people aged ≥40 years (including primary open-angle glaucoma [POAG] in 3.9%, primary angle-closure glaucoma in 0.6%, and secondary glaucoma in 0.5%), and estimates the prevalence of ocular hypertension (OHT) at 0.8% [5, 6].

Elevated intraocular pressure (IOP) and OHT are major modifiable risk factors in the onset and worsening of glaucoma [1, 7]; as such, lowering IOP represents the current standard of care [8–10]. Topical prostaglandin F (FP) receptor agonists (e.g., latanoprost) are the most common first-line treatment, though several other IOP-lowering therapies are available. These include prostaglandin E_2_ receptor EP2 subtype (EP2) agonists (e.g., omidenepag isopropyl, with demonstrable long-term efficacy and safety in normal tension glaucoma [11]), β-adrenergic blockers, α-adrenergic agonists, carbonic anhydrase inhibitors (CAIs), and Rho kinase (ROCK) inhibitors. Current guidelines recommend that whenever an IOP-lowering treatment fails to reduce IOP to target levels, switching to another monotherapy or initiating multi-drug combination therapy should be considered [8–10]. However, IOP-lowering monotherapies are often insufficient to achieve target IOP levels [12], and multi-drug combinations are associated with poor patient adherence [13].

In August 2025, sepetaprost—a novel dual agonist of FP and prostaglandin E_2_ receptor EP3 subtype (EP3) receptors [14]—received manufacturing and marketing approval in Japan for the treatment of glaucoma and OHT [15]. Based on preclinical findings that the IOP-lowering effects of FP receptor agonists may be mediated in part by EP3 receptors [16–18], it was hypothesised that dual activation of these pathways with sepetaprost may reduce IOP beyond FP receptor stimulation alone. To that end, the efficacy and safety of sepetaprost have been evaluated in several clinical trials [19–24], including a phase 2b, randomised, dose-finding study in patients with POAG or OHT [25]. In that study, once-daily administration of sepetaprost 0.002% was found to be the optimal dose, based on comparable IOP-lowering efficacy and safety versus latanoprost 0.005% [25]. In light of these findings, the present phase 3 ANGEL-J1 study aimed to evaluate the efficacy and safety of sepetaprost versus latanoprost for the treatment of patients with POAG or OHT.

## Patients and methods

### Study design

ANGEL-J1 was a phase 3, multicentre, randomised, investigator-masked, active-controlled, parallel-group, non-inferiority study, conducted at 49 clinical sites in Japan from August 2022 to April 2023. ANGEL-J1 was compliant with the Declaration of Helsinki, the Act on Pharmaceuticals and Medical Devices, and Japanese Good Clinical Practice. The protocol was approved by the institutional review boards of participating sites prior to study commencement, and all patients provided written informed consent. ANGEL-J1 is registered with ClinicalTrials.gov (NCT05495061) and the Japanese Registry of Clinical Trials (jRCT2031220267).

### Study population

Eligible patients were adults aged ≥18 years with POAG or OHT OU, or POAG in one eye and OHT in the other. Included patients also had a corrected visual acuity (VA) of +0.60 logarithm of the minimum angle of resolution (logMAR) units or better (decimal VA ≥0.3), a central corneal thickness of 480–620 µm, and an anterior chamber angle of Shaffer grade ≥2 OU. At the baseline study visit (described below), IOP at three time points (9:00, 13:00, and 17:00) was required to be ≥22 mmHg in at least one eye and ≤34 mmHg in both. Key exclusion criteria included any prior history of ocular surgery to reduce IOP, any ocular surgery or laser treatment within 90 days of screening, or any change to topical or systemic treatments known to affect IOP within 30 days of screening. Full study eligibility criteria are available upon request.

One eye per patient was designated the study eye, defined as the eye that met all eligibility criteria at the baseline study visit. If both eyes were eligible, the eye with higher mean diurnal IOP at baseline was selected. If both eyes had the same mean diurnal IOP, the right eye was designated the study eye.

### Study procedures

The study comprised a washout period of up to 35 days, followed by a 3-month treatment period. After an initial screening visit, eligible patients entered a washout period where all prior IOP-lowering treatments were discontinued. The duration of the washout period for each patient was based on the prior treatment(s) received, plus up to 7 additional days: 7 days for miotics and CAIs; 14 days for α-adrenergic agonists and α/β-adrenergic agonists; and 28 days for α-adrenergic antagonists, β-adrenergic blockers, prostaglandin analogues, and ROCK inhibitors. For patients receiving multiple prior treatments, the longest washout period was applied; treatment-naïve patients could progress to the next study visit without a washout period.

On day 1 of the treatment period, patients were randomised 1:1 to receive either sepetaprost 0.002% or latanoprost 0.005% ophthalmic solution. Patients were instructed to apply one drop of sepetaprost or latanoprost to both eyes, once daily at 21:00 (±60 minutes) for 3 months. Patients were randomised to their assigned treatment using a Latin square (block size 4), generated by the treatment allocation manager using a central computer system. The code used for treatment allocation was sealed and stored. Sepetaprost and latanoprost ophthalmic solutions were contained in non-identical bottles, but were packaged in identical boxes to ensure that investigators were masked to treatment assignments.

After baseline, patients attended study visits at week 2 (day 15±3), week 4 (day 29±3), and month 3 (day 91±7). Key ocular assessments at each visit included VA tests, slit-lamp microscopy, and IOP measured at three time points (9:00, 13:00, and 17:00; all ±60 minutes) using a Goldmann applanation tonometer. IOP was calculated as the mean of two consecutive IOP measurements at each time point; if the two measurements differed by ≥3 mmHg, a third measurement was taken and IOP was calculated as the median. Adverse events (AEs) were monitored throughout the study and coded according to Medical Dictionary of Regulatory Activities thesaurus terms (MedDRA version 25.0).

### Outcome measures

Efficacy outcomes were assessed in the study eye for each patient. The primary efficacy endpoint was change in mean diurnal IOP from baseline at week 4, where diurnal IOP was calculated as the mean of the 9:00, 13:00, and 17:00 IOP measurements at each visit. A key secondary efficacy endpoint was change in IOP (at 9:00, 13:00, and 17:00) from baseline at week 2, week 4, and month 3.

Other secondary efficacy outcomes reported herein include changes in mean diurnal IOP from baseline at week 2 and month 3; the proportion of patients who achieved mean diurnal IOP reductions of ≥20%, ≥25%, or ≥30% from baseline at each visit; and the proportion of patients who achieved mean diurnal IOP ≤18 mmHg at each visit. Safety outcomes included the incidence and severity of AEs and adverse drug reactions (ADRs; defined as any AE related to study treatment, as determined by the investigators).

### Statistical analysis

A sample size of 150 patients per treatment group (i.e., N=300) would provide 90% power to detect the non-inferiority of sepetaprost versus latanoprost for the primary endpoint. This sample size calculation used a non-inferiority margin of 1.5 mmHg and assumed a standard deviation (SD) of 3.8 mmHg (derived from the phase 2b dose-finding study of sepetaprost in POAG or OHT [25]), a two-sided significance level of 5%, and a dropout rate of 10%.

Efficacy analyses were based on the full analysis set, defined as all randomised patients who received ≥1 dose of study treatment, and who had evaluable IOP measurements at baseline and ≥1 study visit thereafter. Sensitivity analyses were performed to evaluate the primary endpoint in the per-protocol set, defined as patients in the full analysis set who met all study eligibility criteria; had evaluable IOP at three time points (9:00, 13:00, and 17:00) at baseline or ≥1 study visit thereafter; demonstrated ≥75% adherence to study treatment; and had no protocol deviations that may impact treatment efficacy.

The primary and key secondary endpoints were evaluated using mixed model for repeated measures (MMRM) analyses, which included treatment, visit, and treatment-by-visit interaction as fixed effects, baseline mean diurnal IOP as a covariate (or baseline IOP at each time point, as applicable), and subject as a random effect. Within-subject errors were modelled using an unstructured covariance matrix, and analyses were based on observed data with no imputation for missing values. MMRM analyses were summarised using least-squares (LS) means for the primary and key secondary endpoints, standard errors (SE), differences in LS means between treatment groups, and associated 95% confidence intervals (CI). Three hypotheses were considered using a fixed sequence method to control the overall type 1 error rate:

1. Non-inferiority of sepetaprost versus latanoprost for the primary endpoint (non-inferiority margin of 1.5 mmHg);
2. Non-inferiority of sepetaprost versus latanoprost for the key secondary endpoint (non-inferiority margin of 1.5 mmHg for all time points and 1.0 mmHg for >50% of all time points), and;
3. Superiority of sepetaprost versus latanoprost for the primary endpoint.

The secondary endpoints of change in mean diurnal IOP from baseline at week 2 and month 3 were evaluated using MMRM analyses as described above. For mean diurnal IOP response rates (i.e., categorical variables for which MMRM is not applicable and other repeated measures models [e.g., generalised linear mixed models] are complex for secondary endpoints), the number and proportion of patients with each response were summarised by treatment group and study visit. Missing IOP values were imputed using last observation carried forward methodology; differences in IOP response rates between treatment groups were presented with 95% CIs and tested for significance using Fisher’s exact test.

Safety outcomes were evaluated in the safety analysis set, defined as all randomised patients who received ≥1 dose of study treatment. Safety was assessed through descriptive summaries of AEs and ADRs reported throughout the study period. Statistical analyses were performed using SAS version 9.4 or later (SAS Institute).

## Results

### Study population

**Fig. 1.**
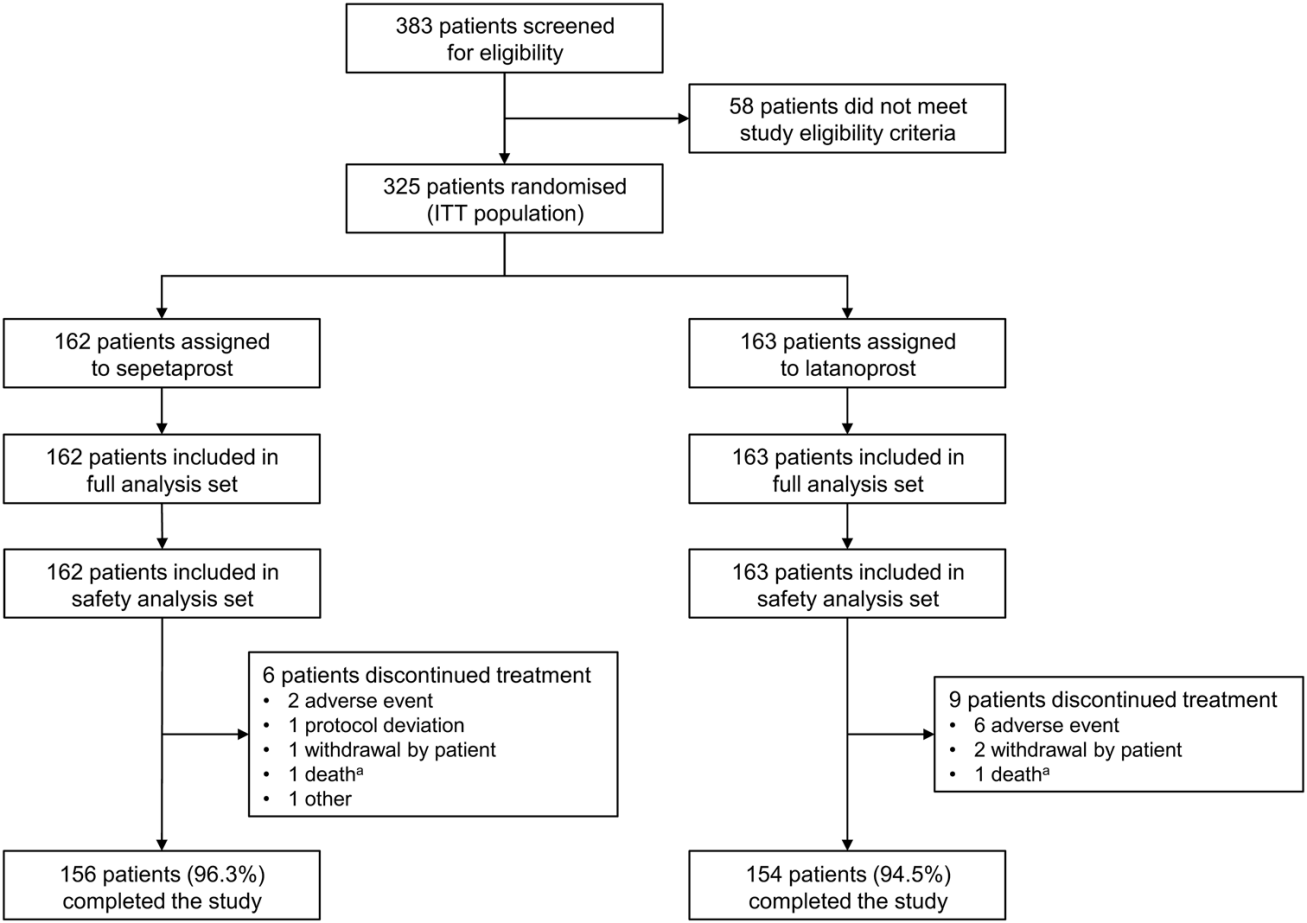
Patient disposition. ^a^Not related to study treatment or adverse events. ITT, intention-to-treat.

In total, 383 patients with POAG or OHT were screened for eligibility in ANGEL-J1 (Fig. 1). After exclusions, 325 patients comprised the intention-to-treat population and were randomised to receive sepetaprost (n=162) or latanoprost (n=163). All 325 patients in the intention-to-treat population were subsequently included in the full and safety analysis sets to evaluate efficacy and safety outcomes, respectively. Of these, 154 patients (95.1%) in the sepetaprost group and 156 (95.7%) in the latanoprost group were included in the per-protocol set for sensitivity analyses.

Baseline patient demographics and clinical characteristics were generally balanced between treatment groups (Table 1). Overall, mean±SD age at baseline was 62.7±13.0 years, 61.5% of patients were female, and 45.2% had a primary diagnosis of POAG. Mean diurnal IOP (mean±SD) in the study eye at baseline was 23.3±1.5 mmHg in the sepetaprost group, versus 23.1±1.2 mmHg in the latanoprost group.

**Table 1:** Baseline patient demographics and clinical characteristics (full analysis set)

	Sepetaprost (n = 162)	Latanoprost (n = 163)	Total (N = 325)
Age, years			
Mean±SD	62.6±12.6	62.7±13.4	62.7±13.0
Median (range)	65.0 (23, 88)	65.0 (27, 90)	65.0 (23, 90)
<65, n (%)	78 (48.1)	81 (49.7)	159 (48.9)
≥65, n (%)	84 (51.9)	82 (50.3)	166 (51.1)
Female sex, n (%)	100 (61.7)	100 (61.3)	200 (61.5)
Primary diagnosis, n (%)			
OHT	88 (54.3)	90 (55.2)	178 (54.8)
POAG	74 (45.7)	73 (44.8)	147 (45.2)
Prior IOP-lowering therapies, n (%)			
Prostamide or prostaglandin analogue	66 (40.7)	65 (39.9)	131 (40.3)
β-adrenergic blocker	34 (21.0)	36 (22.1)	70 (21.5)
Carbonic anhydrase inhibitor	14 (8.6)	16 (9.8)	30 (9.2)
α-adrenergic agonist	11 (6.8)	5 (3.1)	16 (4.9)
Rho kinase inhibitor	2 (1.2)	0	2 (0.6)
Miotic agent	0	0	0
Other	11 (6.8)	12 (7.4)	23 (7.1)
None	64 (39.5)	66 (40.5)	130 (40.0)
Mean diurnal IOP, mmHg			
Mean ± SD	23.3±1.5	23.1±1.2	23.2±1.4
Median (range)	22.8 (22.0, 30.3)	22.8 (22.0, 31.5)	22.8 (22.0, 31.5)
IOP at 9:00, mmHg			
Mean ± SD	23.6±1.8	23.3±1.5	23.4±1.7
Median (range)	23.0 (22.0, 34.0)	23.0 (22.0, 32.0)	23.0 (22.0, 34.0)
IOP at 13:00, mmHg			
Mean ± SD	23.3±1.6	23.1±1.4	23.2±1.5
Median (range)	23.0 (22.0, 32.0)	23.0 (22.0, 30.5)	23.0 (22.0, 32.0)
IOP at 17:00, mmHg			
Mean ± SD	23.1±1.5	23.0±1.3	23.1±1.4
Median (range)	22.5 (22.0, 29.5)	22.5 (22.0, 32.0)	22.5 (22.0, 32.0)
CCT, µm			
Mean ± SD	553.6±33.1	560.7±35.4	557.2±34.4
Median (range)	555.0 (483, 617)	563.0 (483, 620)	559.0 (483, 620)
VA, LogMAR units			
Mean ± SD	−0.04±0.12	−0.04±0.09	−0.04±0.11
Median (range)	−0.08 (−0.30, 0.52)	−0.08 (−0.18, 0.40)	−0.08 (−0.30, 0.52)
Degree of angle closure, n (%)			
Shaffer grade 2	3 (1.9)	2 (1.2)	5 (1.5)
Shaffer grade 3–4	159 (98.1)	161 (98.8)	320 (98.5)

CCT, central corneal thickness; IOP, intraocular pressure; LogMAR, logarithm of the minimum angle of resolution; OHT, ocular hypertension; POAG, primary open-angle glaucoma; SD, standard deviation; VA, visual acuity

### Efficacy outcomes

**Fig. 2.**
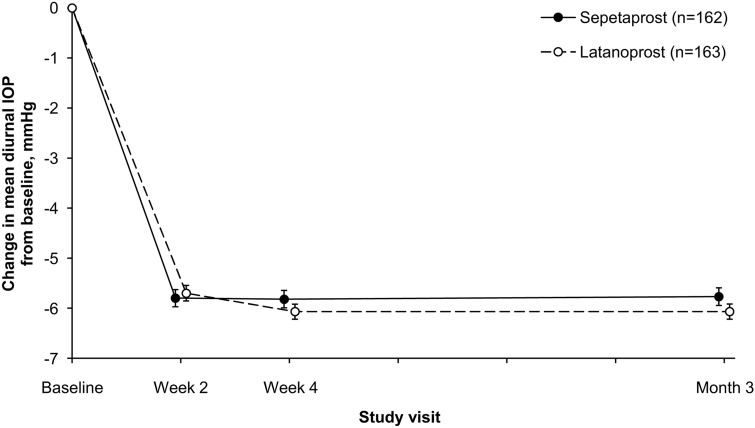
Change in mean diurnal IOP from baseline over time (full analysis set). Data are mean ± standard error. Analyses are based on observed data at each time point, with no imputation for missing values. IOP, intraocular pressure.

The observed change in mean diurnal IOP over time is presented in Fig. 2, and the results of MMRM analyses for this outcome are summarised in Supplementary Table 1. ANGEL-J1 met its primary endpoint, demonstrating non-inferior reductions in mean diurnal IOP from baseline at week 4 with sepetaprost versus latanoprost. At week 4, the LS mean (±SE) change in mean diurnal IOP from baseline was −5.77±0.16 mmHg in the sepetaprost group versus −6.10±0.16 mmHg with latanoprost, yielding a LS mean difference of 0.32 mmHg (95% CI, −0.12, 0.77). Since the upper limit of the 95% CI for the adjusted mean difference did not exceed 1.5 mmHg, the non-inferiority of sepetaprost versus latanoprost was established (hypothesis 1). Sensitivity analyses yielded comparable estimates for the primary endpoint in the per-protocol set, thus confirming that the results of the primary analyses were robust (Supplementary Table 2). In the full analysis set, LS mean (±SE) changes in mean diurnal IOP from baseline were also comparable between the sepetaprost and latanoprost groups at both the week 2 (−5.78±0.16 vs −5.73±0.16 mmHg, respectively) and month 3 (−5.76±0.16 vs −6.07±0.16 mmHg) study visits.

MMRM analyses for the key secondary endpoint are presented in Table 2. LS mean changes in IOP from baseline ranged from −5.62 to −5.95 mmHg across time points and study visits in the sepetaprost group, and from −5.58 to −6.21 mmHg in the latanoprost group (LS mean differences between groups ranged from −0.20 to +0.47 mmHg). Since the upper limits of the 95% CIs for adjusted mean differences did not exceed 1.5 mmHg at all time points, and did not exceed 1.0 mmHg for >50% of all time points, the non-inferiority of sepetaprost versus latanoprost was established (hypothesis 2). Based on this result, hypothesis 3 was subsequently tested; however, since the upper limit of the 95% CI for the adjusted mean difference between sepetaprost and latanoprost for the primary endpoint was ≥0 mmHg, superiority was not demonstrated (Supplementary Table 1).

**Table 2:** Change in IOP from baseline over time (full analysis set)

Change in IOP from baseline, mmHg	Week 2		Week 4		Month 3	
	Sepetaprost (n = 162)	Latanoprost (n = 163)	Sepetaprost (n = 162)	Latanoprost (n = 163)	Sepetaprost (n = 162)	Latanoprost (n = 163)
9:00, n	160	162	158	160	156	154
LS mean ± SE	−5.62±0.18	−5.58±0.18	−5.73±0.17	−6.05±0.17	−5.62±0.18	−6.03±0.18
Difference ± SE	−0.04±0.25		0.32±0.25		0.41±0.26	
95% CI	−0.53, 0.45		−0.16, 0.80		−0.09, 0.92	
13:00, n	159	162	158	160	156	153
LS mean ± SE	−5.73±0.19	−5.87±0.19	−5.74±0.18	−6.21±0.18	−5.70±0.18	−6.16±0.18
Difference ± SE	0.15±0.26		0.47±0.25		0.46±0.25	
95% CI	−0.37, 0.67		−0.02, 0.97		−0.04, 0.96	
17:00, n	159	162	158	160	156	154
LS mean±SE	−5.95±0.18	−5.75±0.17	−5.83±0.17	−6.06±0.17	−5.93±0.18	−6.07±0.18
Difference ± SE	−0.20±0.25		0.23±0.25		0.13±0.25	
95% CI	−0.69, 0.28		−0.25, 0.71		−0.36, 0.63	

Data are based on MMRM analyses, which included treatment, visit, and treatment-by-visit interaction as fixed effects, baseline IOP (at 9:00, 13:00, or 17:00) as a covariate, and subject as a random effect. Within-subject errors are modelled using an unstructured covariance matrix. Analyses are based on observed data at each time point, with no imputation for missing values

*CI* confidence interval, *IOP* intraocular pressure, *LS* least-squares, *MMRM* mixed model for repeated measures, *SE* standard error

The proportions of patients who achieved a ≥20% reduction in mean diurnal IOP from baseline at week 4, and a mean diurnal IOP ≤18 mmHg at week 4, were statistically lower in the sepetaprost group versus latanoprost (*p*=0.015 and *p*=0.002, respectively; Supplementary Table 3). However, the proportions of patients who achieved these responses at week 2 or month 3 were not significantly different between treatment groups, and there were no significant between-group differences in the proportions of patients who achieved ≥25% or ≥30% reductions in mean diurnal IOP from baseline at any study visit.

### Safety outcomes

During the treatment period, AEs were reported in 86 patients (53.1%) receiving sepetaprost and 68 (41.7%) receiving latanoprost (Table 3). Most AEs were ocular events, occurring in 68 patients (42.0%) in the sepetaprost group and 53 (32.5%) in the latanoprost group. The majority of AEs were mild in severity; the incidence of moderate AEs was 1.9% (3 patients) in the sepetaprost group versus 3.7% (6 patients) in the latanoprost group, and no severe AEs were reported.

**Table 3:** Summary of AEs and ADRs during the treatment phase (safety analysis set)

n (%)	Sepetaprost (n = 162)	Latanoprost (n = 163)	Total (N = 325)
Patients with any event			
AE	86 (53.1)	68 (41.7)	154 (47.4)
Mild AE	83 (51.2)	62 (38.0)	145 (44.6)
Moderate AE	3 (1.9)	6 (3.7)	9 (2.8)
Severe AE	0	0	0
SAE	0	1 (0.6)	1 (0.3)
ADR	59 (36.4)	33 (20.2)	92 (28.3)
Ocular ADR	59 (36.4)	33 (20.2)	92 (28.3)
Non-ocular ADR	1 (0.6)	0	1 (0.3)
Serious ADR	0	0	0
AE leading to treatment discontinuation	2 (1.2)	6 (3.7)	8 (2.5)
AE resulting in death	0	0	0

ADRs in ≥2% of patients^a^			
Conjunctival hyperaemia	48 (29.6)	14 (8.6)	62 (19.1)
Mild	46 (28.4)	14 (8.6)	60 (18.5)
Moderate	2 (1.2)	0	2 (0.6)
Eye irritation	3 (1.9)	7 (4.3)	10 (3.1)
Punctate keratitis	2 (1.2)	6 (3.7)	8 (2.5)
Erythema of eyelid	4 (2.5)	0	4 (1.2)

Patients with multiple events in each row are counted only once, at the maximum severity

^a^Unless otherwise stated, ADRs were mild in severity. No severe ADRs were reported

*ADR* adverse drug reaction, *AE* adverse event, *SAE* serious adverse event

ADRs were reported in 59 patients (36.4%) receiving sepetaprost and 33 (20.2%) receiving latanoprost (Table 3). The most common ADR in both groups was conjunctival hyperaemia, with an incidence of 29.6% (48 patients) in the sepetaprost group and 8.6% (14 patients) in the latanoprost group. Except for moderate conjunctival hyperaemia in 2 patients (1.2%) receiving sepetaprost, all other conjunctival hyperaemia ADRs were mild in severity. The next most common ADR in the sepetaprost group was eyelid erythema (4 patients [2.5%]; all mild), and the next most common ADRs in the latanoprost group were eye irritation (7 patients [4.3%]; all mild) and punctate keratitis (6 patients [3.7%]; all mild). All other ADRs occurred in <2% of patients in each treatment group, and no serious ADRs were reported.

## Discussion

The phase 3 ANGEL-J1 study demonstrates the efficacy and tolerability of sepetaprost, a novel dual FP/EP3 receptor agonist, for the treatment of POAG or OHT. ANGEL-J1 met its primary endpoint, showing that sepetaprost offered reductions in mean diurnal IOP that were non-inferior to latanoprost at week 4 and then sustained over 3 months. The non-inferiority of sepetaprost versus latanoprost for changes in IOP over time (a key secondary endpoint) was also established, and mean diurnal IOP response rates were generally comparable between groups. Sepetaprost demonstrated an acceptable safety profile overall; most AEs and ADRs were mild in severity, and no severe or serious ADRs were reported. Together, these data suggest that sepetaprost, with its novel mechanism of dual FP/EP3 receptor activation, may represent a new IOP-lowering treatment option for patients with POAG or OHT.

The results of our efficacy analyses are consistent with previous studies that have evaluated the IOP-lowering effects of sepetaprost in patients with POAG or OHT [21–25]. In the present study, LS mean reductions in mean diurnal IOP from baseline were sustained at approximately 5.8 mmHg over 3 months of once-daily sepetaprost treatment, which is comparable with mean reductions of 6.6–6.9 mmHg reported over the same time period in the previous phase 2b ANGEL study [25]. LS mean reductions in IOP with sepetaprost ranged from 5.6–6.0 mmHg across time points and visits in this study (vs means of 6.1–7.2 mmHg in ANGEL [25]), 75% of sepetaprost-treated patients achieved ≥20% reductions in mean diurnal IOP at month 3 (vs 84%), and 66% of those achieved a mean diurnal IOP ≤18 mmHg at month 3 (vs 61%). Mean baseline IOP was numerically lower in this study versus the previous ANGEL study (which included patients from the United States, who had higher mean IOP than Japanese patients at baseline [25]); as a result, IOP reductions in this Japanese study were numerically smaller for most efficacy endpoints. Furthermore, although significant between-group differences were detected for some secondary outcomes (i.e., the proportion of patients who achieved a ≥20% reduction in mean diurnal IOP from baseline at week 4, or a mean diurnal IOP ≤18 mmHg at week 4), it should be noted that dichotomised endpoints may be sensitive to threshold effects and should be interpreted in conjunction with continuous outcome measures. Indeed, analyses of the primary and key secondary endpoints demonstrate that the IOP-lowering efficacy of sepetaprost over 3 months was non-inferior to latanoprost, one of the most efficacious first-line therapies currently available for the management of glaucoma [26]. Although nocturnal or trough IOP were not evaluated in this study, a recent exploratory study found that mean 24-hour IOP and nocturnal IOP were each numerically lower with sepetaprost versus latanoprost after 3 months of treatment [24]. These data suggest that sepetaprost might have greater potential to reduce IOP fluctuations between doses in patients with POAG or OHT.

The most frequent ADR in this study was conjunctival hyperaemia, an AE commonly associated with prostaglandin analogues and other IOP-lowering therapies for glaucoma [27, 28]. The incidence of conjunctival hyperaemia ADRs was numerically higher in the sepetaprost group versus latanoprost, which likely contributed to the higher incidence of ADRs overall in the sepetaprost group. All other ADRs had an incidence <5% in each group, and all except three (eye irritation and punctate keratitis in the latanoprost group, and eyelid erythema in the sepetaprost group) had an incidence <2%. The majority of ADRs in this study were mild in severity and no serious ADRs were reported, supporting the overall safety and tolerability of sepetaprost in patients with POAG or OHT.

Glaucoma is a chronic disease that requires ongoing management; therefore, a key limitation of the present ANGEL-J1 study was its relatively short follow-up of 3 months. This was addressed in part by the randomised, open-label, phase 3 ANGEL-J2 study, which was conducted in parallel to assess longer-term outcomes of sepetaprost treatment in patients with open-angle glaucoma or OHT [29]. ANGEL-J2 evaluated the safety and IOP-lowering efficacy of sepetaprost, alone or in combination with timolol (a β-adrenergic blocker), over 52 weeks [29]. However, the present results from ANGEL-J1 warrant further long-term comparative studies of sepetaprost versus latanoprost (or another FP receptor agonist), to determine whether dual FP/EP3 receptor activation may offer durable reductions in IOP that are sustained beyond FP activation alone.

In conclusion, the phase 3 ANGEL-J1 study of sepetaprost in POAG or OHT achieved its primary endpoint, demonstrating 4-week reductions in mean diurnal IOP that were non-inferior to latanoprost. This IOP-lowering effect was maintained over 3 months of sepetaprost treatment. Future studies will seek to evaluate longer-term treatment outcomes; however, current data suggest that dual FP/EP3 pathway activation with sepetaprost is a novel, efficacious, and tolerable treatment strategy for the management of POAG or OHT.

## Supplementary Information

Below is the link to the electronic supplementary material.

Supplementary file1 (PDF 61 kb)

## Data Availability

The datasets generated and/or analysed during the current study are not publicly available, but may be requested in accordance with Santen’s data sharing policy.
