# Supplementary material for: Efficacy and safety of sepetaprost in patients with primary open-angle glaucoma or ocular hypertension: results from the randomised, phase 3 ANGEL-J1 study
Source: Jpn J Ophthalmol. 2026 Jun 16;70(4):957–65. doi: 10.1007/s10384-026-01350-3 (PMC13452788; doi:10.1007/s10384-026-01350-3)
Supplement: Supplementary file 1 [file 10384_2026_1350_MOESM1_ESM.pdf]

Supplementary material

Efficacy and safety of sepetaprost in patients with primary open-angle glaucoma or ocular hypertension: results from the randomised, phase 3 ANGEL-J1 study

Authors: Masaru Inatani, Akiyasu Kanamori, Maya Inai, Toshihiro Ikeda, Raymund Angeles

Corresponding author: Raymund Angeles, Santen Inc., 2100 Powell Street, Suite 800, Emeryville, CA 94608, USA. Phone: +1-510-368-9861; E-mail: Raymund.Angeles@santen.com

Supplementary Table 1 Change in mean diurnal IOP from baseline over time (full analysis set)

Change in mean diurnal IOP from baseline, mmHg	Sepetaprost (n=162)	Latanoprost (n=163)
Week 2, n	159	162
LS mean±SE	−5.78±0.16	−5.73±0.16
Difference±SE	−0.05±0.23	
95% CI	−0.51, 0.40	
Week 4, n	158	160
LS mean±SE	−5.77±0.16	−6.10±0.16
Difference±SE	0.32±0.23	
95% CI	−0.12, 0.77	
Month 3, n	156	153
LS mean±SE	−5.76±0.16	−6.07±0.16
Difference±SE	0.32±0.23	
95% CI	−0.14, 0.77	

Data are based on MMRM analyses, which included treatment, visit, and treatment-by-visit interaction as fixed effects, baseline mean diurnal IOP as a covariate, and subject as a random effect. Within-subject errors are modelled using an unstructured covariance matrix. Analyses are based on observed data at each time point, with no imputation for missing values.

CI, confidence interval; IOP, intraocular pressure; LS, least-squares; MMRM, mixed model for repeated measures; SE, standard error.

Supplementary Table 2 Change in mean diurnal IOP from baseline at week 4 (per-protocol set)

**Change in mean diurnal IOP from baseline, mmHg**	**Sepetaprost (n=154)**	**Latanoprost (n=156)**
Week 4, n	152	154
LS mean±SE	−5.74±0.16	−6.12±0.16
Difference±SE	0.38±0.23	
95% CI	−0.07, 0.83	

Data are based on MMRM analyses, which included treatment, visit, and treatment-by-visit interaction as fixed effects, baseline mean diurnal IOP as a covariate, and subject as a random effect. Within-subject errors are modelled using an unstructured covariance matrix. Analyses are based on observed data at each time point, with no imputation for missing values.

CI, confidence interval; IOP, intraocular pressure; LS, least-squares; MMRM, mixed model for repeated measures; SE, standard error.

Supplementary Table 3 Proportion of patients who achieved mean diurnal IOP responses over time (full analysis set)

Mean diurnal IOP response	Week 2		Week 4		Month 3	
	**Sepetaprost (n=162)**	**Latanoprost (n=163)**	**Sepetaprost (n=162)**	**Latanoprost (n=163)**	**Sepetaprost (n=162)**	**Latanoprost (n=163)**
≥20% reduction from baseline, n (%)	112 (69.1)	112 (68.7)	112 (69.1)	132 (81.0)	122 (75.3)	129 (79.1)
Difference, %	0.4		−11.8		−3.8	
95% CI	−9.64, 10.49		−21.17, −2.52		−12.94, 5.28	
P value	1.000		0.015		0.430	
≥25% reduction from baseline, n (%)	73 (45.1)	76 (46.6)	76 (46.9)	81 (49.7)	72 (44.4)	80 (49.1)
Difference, %	−1.6		−2.8		−4.6	
95% CI	−12.40, 9.27		−13.64, 8.08		−15.47, 6.20	
P value	0.824		0.658		0.437	
≥30% reduction from baseline, n (%)	44 (27.2)	50 (30.7)	39 (24.1)	48 (29.4)	39 (24.1)	49 (30.1)
Difference, %	−3.5		−5.4		−6.0	
95% CI	−13.36, 6.34		−14.98, 4.23		−15.63, 3.65	
P value	0.541		0.317		0.261	
Reduced to ≤18 mmHg, n (%)	97 (59.9)	105 (64.4)	95 (58.6)	122 (74.8)	107 (66.0)	123 (75.5)
Difference, %	−4.5		−16.2		−9.4	
95% CI	−15.08, 5.99		−26.30, −6.11		−19.25, 0.43	
P value	0.425		0.002		0.068	

Missing data at each time point were imputed using last observation carried forward methodology; *p* values are based on Fisher's exact tests.

CI, confidence interval; IOP, intraocular pressure.
